# Novel artificial transverse atlantal ligament reconstruction for atlantoaxial instability: a finite element analysis

**DOI:** 10.1186/s13018-026-07077-8

**Published:** 2026-07-04

**Authors:** Qiu Du, Daigui Cao, Jing Peng, Zhiwei Liu, Xu Zhou, Xiaomin Qian, Wenli Ke, Kai Shen

**Affiliations:** Department of Orthopaedics, Chongqing Academy of Medical Sciences, Chongqing General Hospital, Chongqing University, Avenue of Stars 118, Liangjiang New District, Chongqing, 401147 China

**Keywords:** Atlantoaxial instability, Artificial transverse atlantal ligament reconstruction, Finite element analysis, Transverse atlantal ligament rupture

## Abstract

**Background:**

The transverse atlantal ligament (TAL) serves as a crucial anatomical structure in maintaining atlantoaxial stability. Currently, fusion surgery remains the primary therapeutic approach for atlantoaxial instability (AAI) resulting from TAL rupture. However, this surgical intervention inevitably leads to the loss of atlantoaxial mobility and may accelerate degeneration of adjacent segments. Reconstruction of the TAL may emerge as a potential solution to address these clinical challenges. The objective of this study is to evaluate the functionality and stability of an artificial transverse atlantal ligament (ATAL) .

**Methods:**

A normal C0–C3 finite element analysis (FEA) model was developed and validated. Based on this model, additional models were constructed, including a TAL rupture model, a posterior C1–C2 screw and rod fixation model, and four ATAL reconstruction model with varying preload forces. Each model was subjected to anterior-posterior (AP) displacement and range of motion (ROM) tests under a 100 N AP load and a 1.5 Nm rotational moment. The functionality and stability of the C1–C2 segment were analyzed across the models based on the experimental results.

**Results:**

Under a 100 N AP load, the AP displacement of the TAL rupture group increased significantly compared with the intact group. In contrast, the AP displacement of the ATAL reconstruction group without preload slightly decreased compared with the intact group. Under a 1.5Nm rotational moment, in the ATAL reconstruction group without preload, compared to the intact group, the C1–C2 flexion, extension, and lateral bending increased by 2.43%, 5.32%, and 20.82%, respectively, while rotation decreased by 1.18%, and compared to the TAL rupture group, the C1–C2 flexion and rotation slightly decreased by 0.76% and 2.04%, respectively, with no significant changes in extension and lateral bending. Compared to the posterior C1–C2 screw and rod fixation group, the C1–C2 ROM in all directions significantly increased in the ATAL reconstruction group.

**Conclusions:**

ATAL reconstruction enables the atlantoaxial joint to achieve sufficient stability while effectively preserving its ROM in all directions. This approach represents a paradigm shift from the current reliance on fusion techniques, which severely restrict atlantoaxial mobility, offering a novel therapeutic strategy for addressing AAI caused by TAL rupture.

## Introduction

The atlantoaxial joint, being the most flexible joint in the cervical spine, is responsible for approximately 50% of the axial rotational movement of the cervical region [1]. The transverse atlantal ligament (TAL), as the thickest ligament in the atlantoaxial joint, significantly contributes to the stability of the atlantoaxial complex [2]. A rupture in this ligament can cause relative translation of C1–C2, leading to atlantoaxial instability (AAI), which might compress the upper spinal cord, resulting in neurological deficits and even mortality [1, 3]. Current treatment protocols for AAI due to TAL rupture vary depending on the classification type. According to the Dickman classification, rupture of the TAL can be categorized into four types: IA, IB, IIA, and IIB. Type 2 A (tubercle fracture) can achieve bony healing through conservative treatment, whereas types 1 A (midsubstance ligament tears), 1B (osteoperiosteal disruptions), and 2B (tubercle avulsion) require treatment with C1–C2 or C0–C2 fusion surgery, as they do not heal through conservative treatment [3, 4]. However, fusion surgery results in almost complete loss of movement in all directions at the atlantoaxial joint, significantly impacting the patient’s postoperative quality of life [5]. Additionally, fusion surgery shifts pressure to adjacent spinal segments, accelerating the degeneration of these neighboring segments, whereas motion-preserving surgeries can reduce the incidence of adjacent segment degeneration [6].

To preserve the atlantoaxial joint’s range of motion (ROM), enhance postoperative quality of life, and prevent accelerated degeneration of adjacent segments of the atlantoaxial complex, various artificial atlantoaxial joints have emerged [5, 7, 8]. With the iteration of these artificial atlantoaxial joints, their design has become increasingly refined, theoretically capable of replacing the original atlantoaxial joint. However, for the treatment of AAI solely due to the rupture of the TAL, artificial joint replacement might be an excessive treatment, as it is technically demanding and relatively invasive. In particular, the implantation of some artificial joints requires the relatively high-risk transoral approach, further increasing the risk of infection due to prolonged surgery times and larger trauma. Therefore, a more suitable treatment approach is worth exploring.

Recently, Peng Zhang et al. proposed a novel treatment method: using anchors and peroneus longus tendon via a posterior cervical approach to perform “ectopic” reconstruction of the ruptured TAL, validated through cadaver experiments [9]. The researchers mentioned that the limited space around the TAL makes in-situ reconstruction challenging, nearly impossible to achieve, hence the choice of ectopic reconstruction via the posterior cervical approach. However, we hold a different view: firstly, this ectopic reconstruction method requires a larger operating space, making it difficult to control surgical trauma; secondly, the fixed position of the ligament is adjacent to numerous critical structures that are difficult to avoid, posing high surgical risks and technical difficulties. Therefore, a transoral approach using a suture anchor to perform reconstruction at a position approximating the original anatomical location of the TAL might be a superior choice. However, this is also a challenging surgical approach, as Peng Zhang et al. mentioned, the major difficulty being the extremely limited space around the original TAL. To overcome this difficulty, we developed a set of artificial transverse atlantal ligament (ATAL) implantation instruments, which allow the ATAL to pass through the narrow space formed by the lateral mass of atlas, odontoid process, and dura mater, ensuring surgical safety and laying a solid foundation for the reconstruction of the TAL. Nevertheless, as a novel surgical approach, the feasibility of the ATAL reconstruction surgery needs further validation.

We noticed that finite element analysis (FEA) has been widely used in the study of orthopaedic implants, which can analyze stress, kinematics, and dynamics in detail. Compared with cadaver experiments, it has advantages such as convenience, reality, accuracy, and economy, making it a beneficial supplement to cadaver experiments [10]. Therefore, in this study, FEA will be used to verify the function and stability of the atlantoaxial joint after ATAL reconstruction.

## Materials and methods

### Design of implantation instruments

**Fig. 1:**
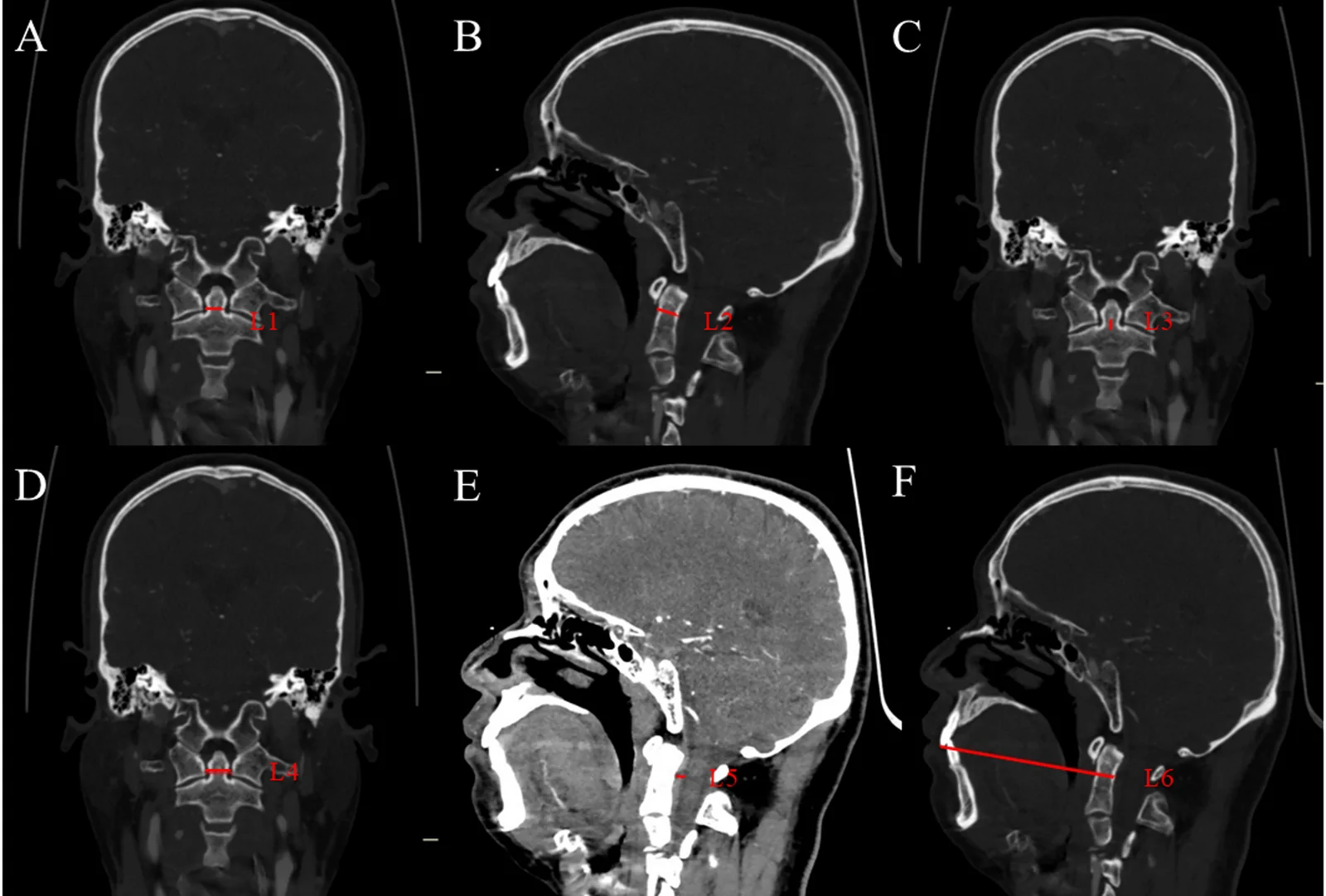
**A** Odontoid neck and surrounding space: the transverse diameter of the odontoid neck (L1); **B** The longitudinal diameter of the odontoid neck (L2); **C** The height of the odontoid neck (L3); **D** The distance between the inferior margins of the lateral masses of the atlas (L4); **E** The distance between the posterior aspect of the odontoid process and the dura mater (L5); **F** The distance between the upper incisors and the posterior margin of the odontoid neck (L6).

**Fig. 2:**
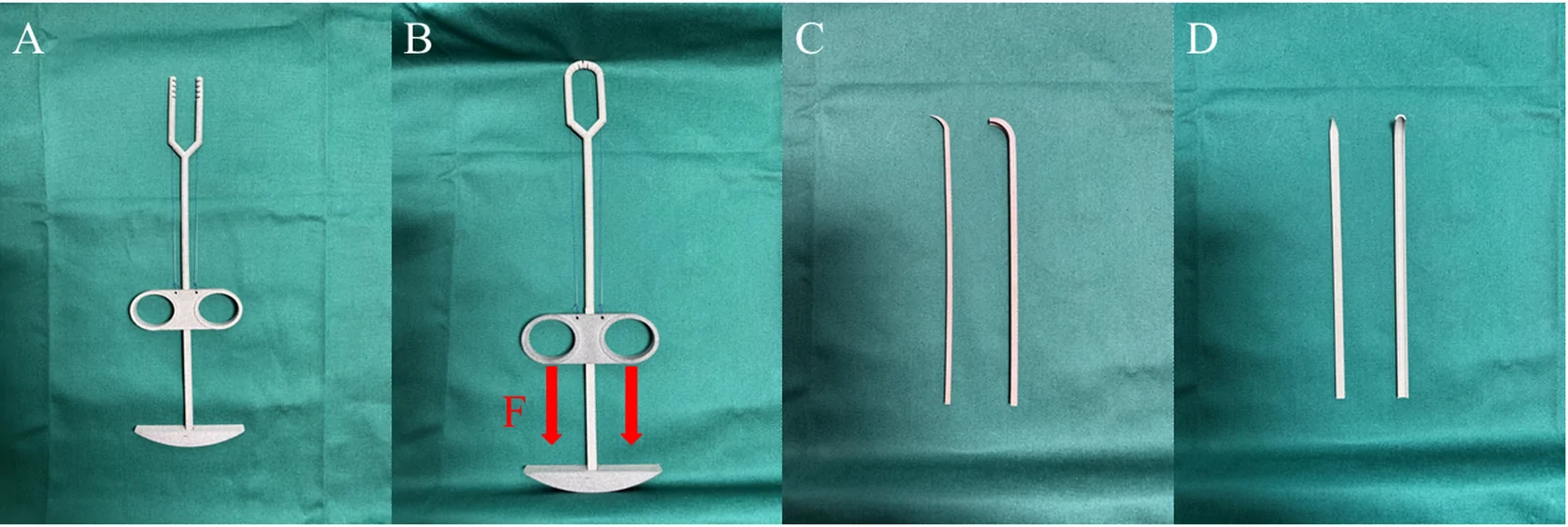
**A** The implanted instruments: the TAL implantation forceps; **B** When the front end of the implantation forceps is subjected to a downward force F (red arrow), a semi-circular closed channel is formed; **C**, **D** The protective guide slot and dissection probe.

The study was conducted in accordance with the Declaration of Helsinki of 1975 as revised in 2013 and was approved by the Medical Ethics Committee of Chongqing General Hospital (No: KY S2022-114-01), with the need for written informed consent waived. All patient details have been de-identified. Computed tomography (CT) images of the head and neck of 40 patients were collected from the radiology imaging data system of Chongqing General Hospital. Patients with a history of cervical trauma, cervical tumors, cervical infections, cervical instability, severe cervical degeneration, cervical deformities, or prior cervical surgery were excluded. The following measurements were taken from the images: the transverse diameter of the odontoid neck (L1), the longitudinal diameter of the odontoid neck (L2), the height of the odontoid neck (L3), the distance between the inferior margins of the lateral masses of the atlas (L4), the distance between the posterior aspect of the odontoid process and the dura mater (L5), and the distance between the upper incisors and the posterior margin of the odontoid neck (L6) (Fig. 1; Table 1). These measurements were used to design the implantation instruments. A 3D model was created using SOLIDWORKS 2021 (Dassault Systemes, Paris, France) and exported in STL format. The file was then imported into a 3D printer for fabrication. The implantation instruments include an ATAL implantation forceps, a protective guide slot, and a dissection probe (Fig. 2).

**Table 1:** Measurement of the size of the odontoid neck bone and the surrounding space (X ± S)

Measurement content	Female (*N* = 20)	Male (*N* = 20)	Mean value	*P*
L1 (mm)	9.15 ± 0.67	9.70 ± 0.92	9.43 ± 0.84	0.038
L2 (mm)	11.25 ± 1.02	12.05 ± 0.83	11.65 ± 1.00	0.010
L3 (mm)	5.20 ± 0.84	5.50 ± 1.00	5.35 ± 0.89	0.294
L4 (mm)	14.65 ± 1.63	15.35 ± 1.73	15.00 ± 1.69	0.195
L5 (mm)	4.45 ± 0.96	4.60 ± 1.05	4.53 ± 0.99	0.637
L6 (mm)	98.55 ± 4.96	104.40 ± 4.12	101.48 ± 5.39	< 0.001

**Fig. 3:**
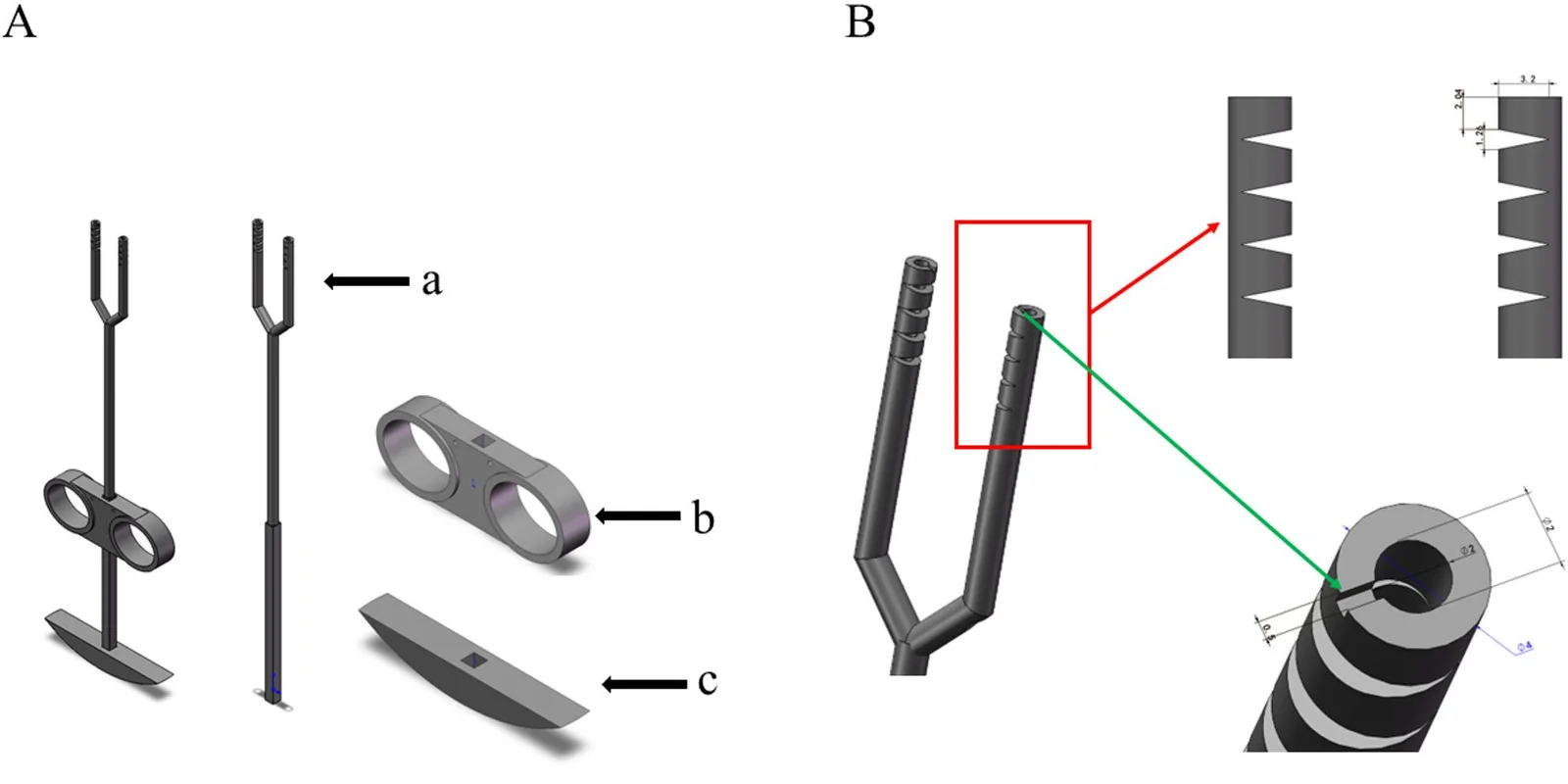
**A** 3D illustration of the implant instrument: the main body component (**a**), the traction component (**b**) and the handle component (**c**) of the artificial TAL implantation forceps; **B** Close-up detail of the front end of the artificial TAL implantation forceps: the traction line is fixed between the top notch of the implantation forceps (green arrow) and the traction component (**b**), transmitting the downward force applied by the user, causing the serrated structure at the front end to bend and form a semi-circular channel (red box).

The ATAL implantation forceps consist of three main components: the main body (a), the traction component (b), and the handle component (c) (Fig. 3A). The user grips the handle component (c) and applies downward force to the traction component (b). This causes the traction component (b) to slide without rotating on the square rod at the lower end of the main body (a). The traction line, fixed between the top inner notches of the main body (a) and the traction component (b), transmits tension to the flexible channel at the front end of the main body (a). Under this tension, the flexible channel gradually bends to form a semicircular channel that can snugly encircle the odontoid neck (Fig. 3B). This design allows the channel to encircle the odontoid neck with minimal space occupation, and the internal diameter of the channel is 2 mm, allowing a guidewire to pass through.

### Simulation of the surgical procedure for ATAL reconstruction

**Fig. 4:**
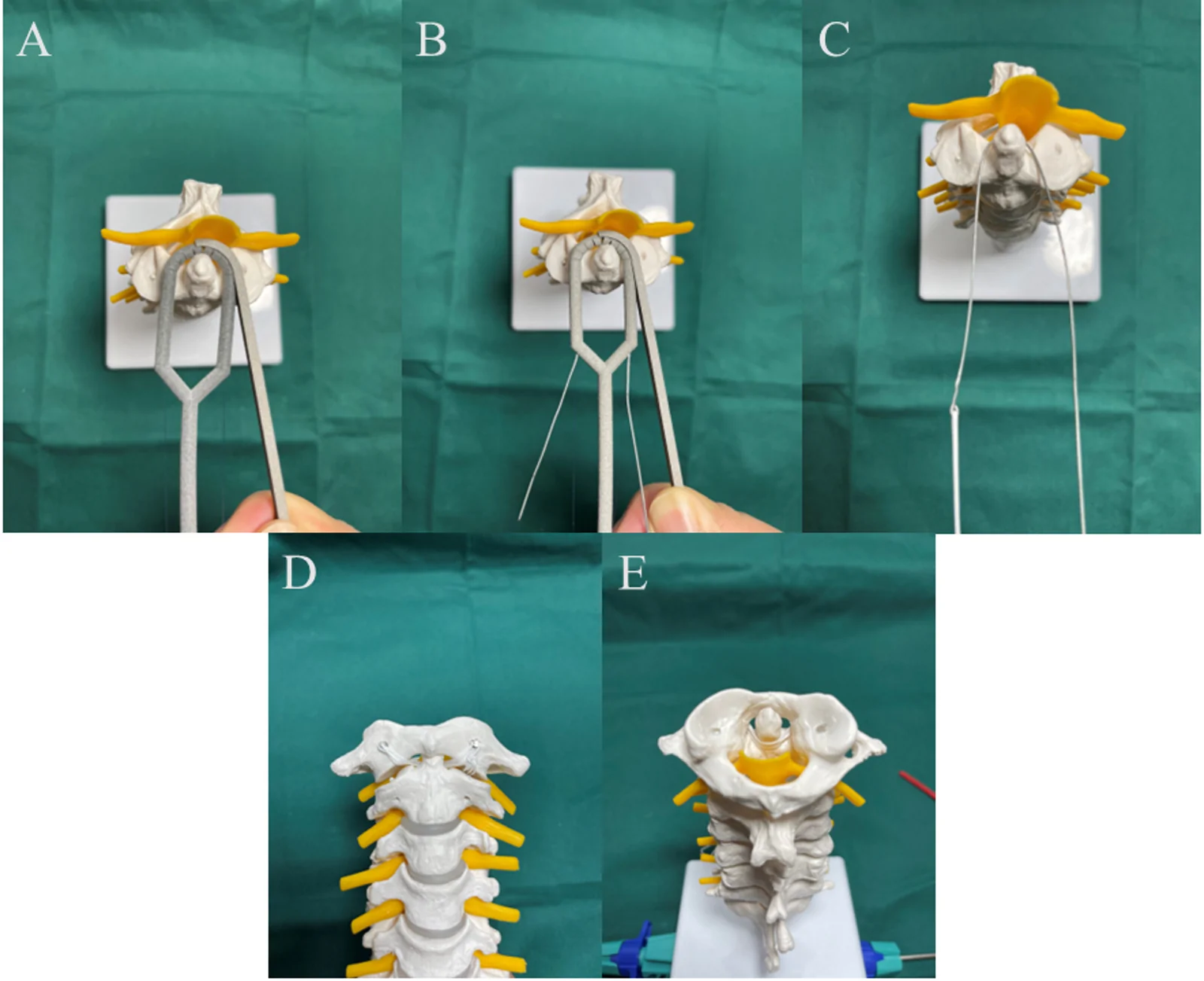
**A** The implantation process of the artificial TAL: the implantation forceps, protected by the protective guide slot, envelop the neck of the dens process to form a passage; **B** The guide wire traverses the passage around the neck of the dens process; **C** After withdrawing the implantation forceps, the ultrabraid suture is threaded through the guide wire around the neck of the dens process; **D**-**E** The ultrabraid suture is secured to both lateral masses of the atlas using anchors.

The classic transoral approach is employed for the reconstruction of the TAL. After positioning, anesthesia, sterilization, and other preoperative preparations are completed, an incision is made along the midline of the posterior pharyngeal wall using a scalpel, starting from the anterior tubercle of the atlas and extending downward to the level of C2. At this point, the longus colli muscles are visible and are separated along the midline. Retractors are used to gently retract the anterior soft tissues of C1 and C2 to the sides, exposing the lateral masses of the atlas and the body of the axis. An ultrasonic bone scalpel is carefully used to abrade the medial and inferior edge of the lateral mass of the atlas, creating a gap of approximately 4 mm between it and the dens process. Finally, with the coordinated use of the implantation instruments, the ATAL is maneuvered around the neck of the dens process and secured with anchors to the lateral masses of the atlas. The specific procedure is as follows (Fig. 4):

1. Using a dissector probe, appropriate soft tissue around the neck of the dens process is gently dissected to create a potential pathway for the protective guide slot to pass through;
2. under the protection of the guide slot, the implantation forcep closely adhere to and navigate around the neck of the dens process, forming a safe semicircular channel for the guide wire to pass through;
3. after the guide wire passes through the channel, the implantation forcep is withdrawn;
4. guided by the wire, the ATAL is maneuvered around the neck of the dens process and secured with two anchor screws to the lateral masses of the atlas.

### FEA model development

**Fig. 5:**
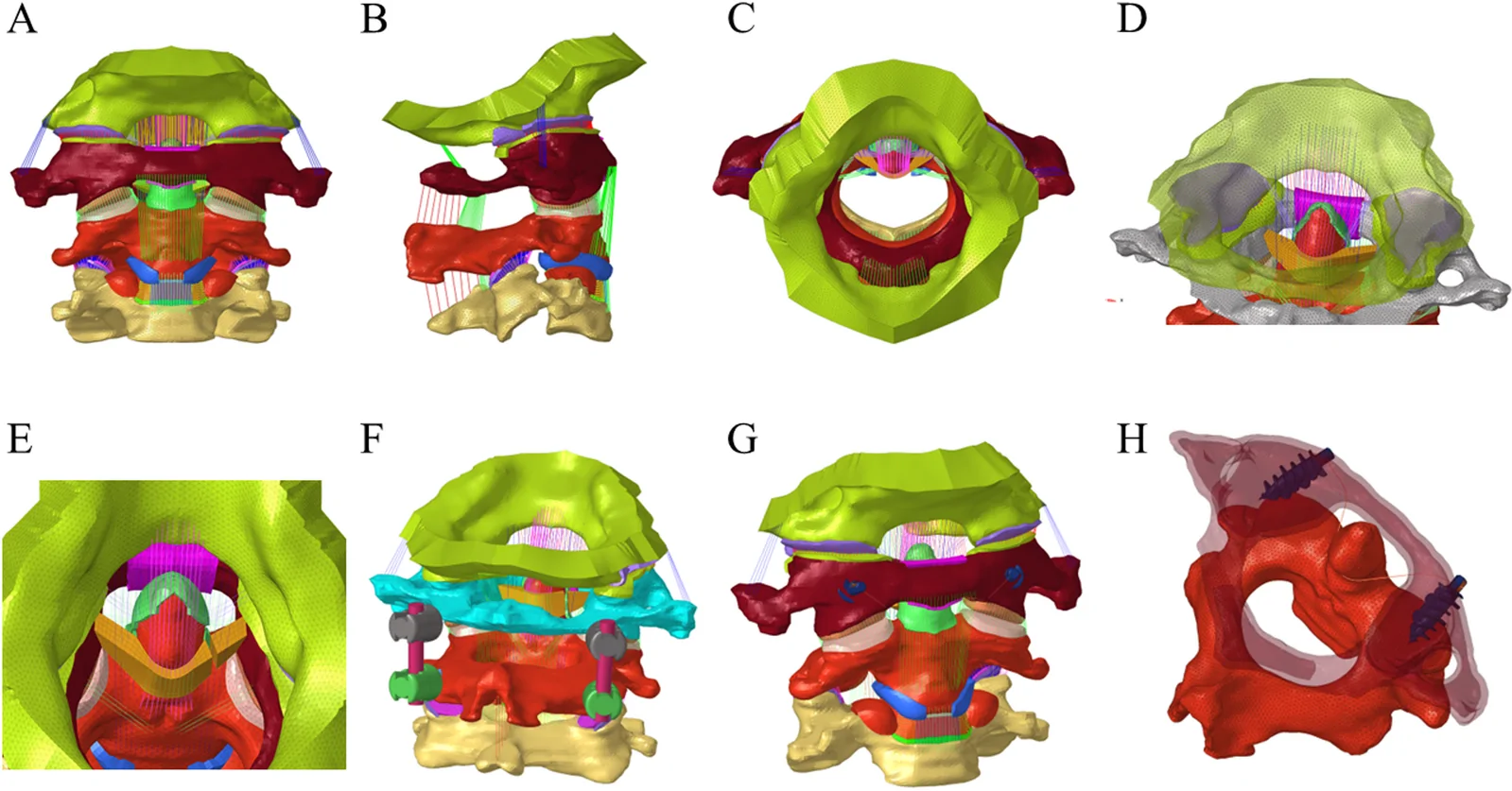
**A**-**C** Structural normal C0-C3 model and extended models: the intact model; **D** The FEA model with the complete TAL; **E** TAL rupture model; F Posterior C1-C2 screw and rod fixation model; **G**–**H** Artificial TAL reconstruction model.

A 26-year-old healthy female volunteer (height: 165 cm, weight: 58 kg) was included in this study. Based on radiological imaging data at 1.0 mm slice thickness from CT (Philips, Amsterdam, Netherlands) scans of the head and neck, conditions such as cervical trauma, deformities, tumors, infections, instability, and severe degeneration were excluded. The CT images of the C0–C3 segments, obtained in DICOM format, were imported into Mimics 25 (Materialise, Leuven, Belgium) for preprocessing, including contrast enhancement, to segment the bone tissue. Using Mimics’ 3D reconstruction capabilities, a geometric model of the cervical spine was generated, encompassing the shape and position of each vertebra. The geometric model was then smoothed in 3-matic 17 (Materialise, Leuven, Belgium) to ensure surface continuity and smoothness. The resulting 3D cervical spine model was imported into HyperMesh 2022 (Altair, Michigan, America) for meshing, with a mesh size of 0.75 mm. Both cortical and cancellous bones were meshed using C3D4 tetrahedral elements. The intervertebral disc was divided into the annulus fibrosus (AF), annulus ground substance, nucleus pulposus, and cartilaginous endplate. Due to their significant deformation, the annulus ground substance and nucleus pulposus were meshed with C3D8H hyperelastic elements. The cartilaginous endplate connected to the intervertebral disc was meshed with C3D8 elements, while the AF was modeled using nonlinear spring elements (Spring A) [11]. The cartilage of joint between the dens and the anterior arch of the atlas, as well as between C0–C1 and C1–C2, was meshed with C3D6 elements, with sliding contact defined between the cartilage surfaces and a friction coefficient of 0.1 [12]. Ligaments, including the vertical cruciate (VC), the alar ligament, the lateral atlantooccipital ligament (LAO), the apical ligament, the anterior and posterior longitudinal ligaments, the ligamentum flavum (LF), the capsular ligament (CL), the interspinous ligaments (ISL), the anterior atlantoaxial ligament (AAOM), the posterior atlantoaxial ligament (PAOM), and the tectorial membrane (TM), were modeled using nonlinear spring elements (Spring A) based on their anatomical locations. The TAL was bonded to the lateral mass of the atlas to simulate their fixed relationship. Given its strength and extensive contact with the dens, the TAL was modeled using solid elements, with sliding contact defined between the dens and the ligament, and a friction coefficient of 0.1 [13]. The final model comprised 112,226 nodes and 473,713 elements (Fig. 5).

Building upon the intact model, a TAL rupture model was simulated by severing one side of the TAL. Further, under the guidance of three experienced spinal surgeons, a posterior C1–C2 pedicle screw and rod internal fixation model was created by implanting four 3.0 mm pedicle screws and two 3.5 mm titanium rods to the atlantoaxial joint. Additionally, two 5.0 mm titanium alloy anchors and ultrabraid suture were fixed on both lateral masses of the atlas and the anchors’ tails, respectively, with the ultrabraid sutures looped around the neck of the odontoid process, forming an ATAL implant model (Fig. 5). All material properties were derived from previous studies as shown in Table 2 [11–18].

**Table 2:** Material properties of bones, ligaments, intervertebral discs, pedicle screws, titanium rods, titanium alloy anchors, and ultrabraid sutures

	Element type	Young modulus (MPa)	Passion’s ratio	Material property
Cortical bone	C3D4	12,000	0.3	–
Cancellous bone	C3D4	300	0.3	–
Cartilage of joint	C3D6	23.8	0.3	–
TAL	C3D8	50	0.3	–
Ligaments	SpringA	–	–	nonlinear force-deformation curves
AF	SpringA	–	–	Stress-strain curve
Annulus ground substance	C3D8H	–	–	C10 = 0.133, C01 = 0.0333, D = 0.6
Nucleus	C3D8H	–	–	C10 = 0.12, C01 = 0.09
Cartilaginous endplate	C3D8	23.8	0.3	–
Pedicle screws and titanium rods	C3D8	110,000	0.3	–
Titanium alloy anchors	C3D4	110,000	0.3	–
Ultrabraid suture	T3D2	3000	0.4	–

### Boundary and loading conditions

The established intact model (C0–C3), TAL rupture model, posterior C1–C2 screw and rod fixation model, and ATAL reconstruction model were divided into four groups as follows: (1) intact group; (2) TAL rupture group; (3) posterior C1–C2 screw and rod fixation group; (4) ATAL reconstruction group. Within the ATAL reconstruction group, models were categorized by applying preload forces ranging from 0 N to 30 N in 10 N intervals to the TAL, resulting in four distinct sub-groups. All models underwent analysis within Abaqus 2020 (Dassault System, Paris, France), with a vertical load of 50 N applied at the volume center of C0, simulating head weight [19]. For anterior-posterior (AP) displacement testing, a 100 N load was applied to C1 while restricting all degrees of freedom at the lower surface of C2, recording the translational distance of C1 relative to C2. For ROM testing, nodes at the lower surface of the C3 segment in each model were constrained in all directions. A rotational moment of 1.5Nm was applied to C0 to induce flexion, extension, lateral bending, and axial rotation, and the ROM values for the C0–C1 and C1–C2 segments were recorded.

### Statistical analysis

Statistical analysis was performed using the SPSS 27.0 software (SPSS Inc, Chicago, IL). We conducted parametric or non-parametric tests to validate the normal distribution of “L1, L2, L3, L4, L5 and L6”. Normally distributed metric data were expressed as mean ± standard deviation (SD), and between-group comparisons were performed using independent sample t-tests. Differences were considered statistically significant at *P* < 0.05.

## Results

### Validation of the normal C0-C3 model

A comparison with the data obtained by Panjabi et al. [20–23] for the ROM of C0–C1 and C1–C2 segments under a 1.5 Nm load in cadaveric specimens reveals that the motion ranges for flexion, extension, lateral bending, and axial rotation in the structurally intact group model for both C0–C1 and C1–C2 segments fall within the mean ± SD of the experimental data from the cadaveric specimens (Table 3). This validates the model’s reliability.

**Table 3:** Validation of the FEA model of intact group through comparison with biomechanical experiments conducted by Panjabi et al

	Level	Biomechanical Experiment Moment = 1.5 Nm	Intact model Moment = 1.5 Nm
Flexion	C0–C1	11.20–17.60	14.783
	C1–C2	9.50–15.90	15.746
Extension	C0–C1	11.20–17.60	11.477
	C1–C2	5.50–15.50	13.407
Lateral bending	C0–C1	2.60–8.60	3.029
	C1–C2	3.80–19.60	5.141
Axial rotation	C0–C1	1.00-10.50	9.535
	C1–C2	24.20–46.40	31.294

### AP displacement test

**Fig. 6:**
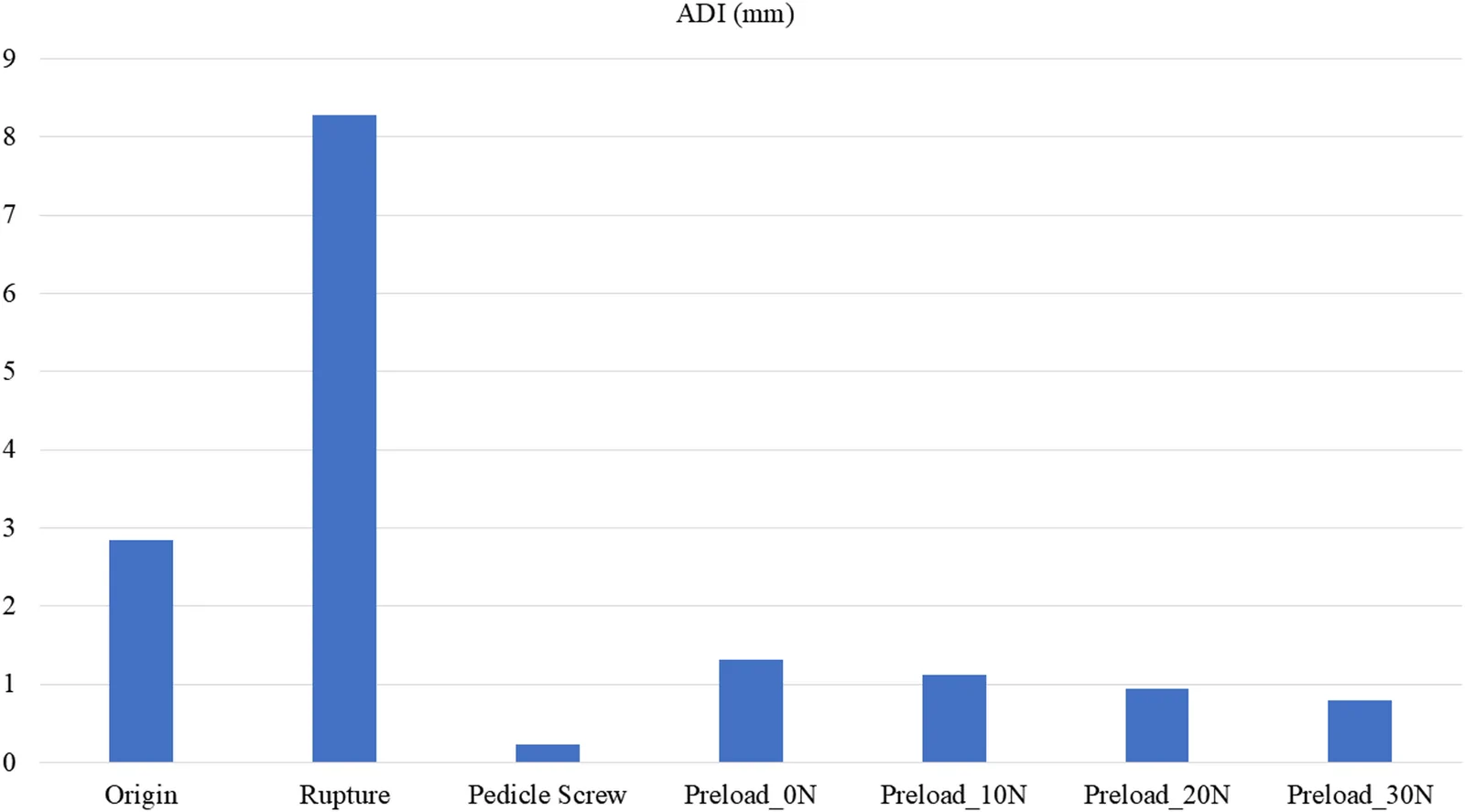
The relative AP translation distances of the C1–C2 for each model under 100 N AP load. ADI, atlantodental interval.

In the ATAL reconstruction group, as the pretension increased incrementally from 0 N to 30 N in 10 N intervals, the AP translation distance gradually decreased, indicating progressively enhanced stability. Compared to the intact group, the AP translation in the TAL rupture group increased significantly by 191.45%. The ATAL reconstruction group without pretension showed an AP reduction of only 53.87%, compared to the intact group; however, it increased the AP by 477.53%, compared to the posterior C1–C2 screw and rod fixation group. Nevertheless, compared to the TAL rupture group, the AP decreased by 84.17%, in the ATAL reconstruction group without pretension (Fig. 6).

### ROM test

**Fig. 7:**
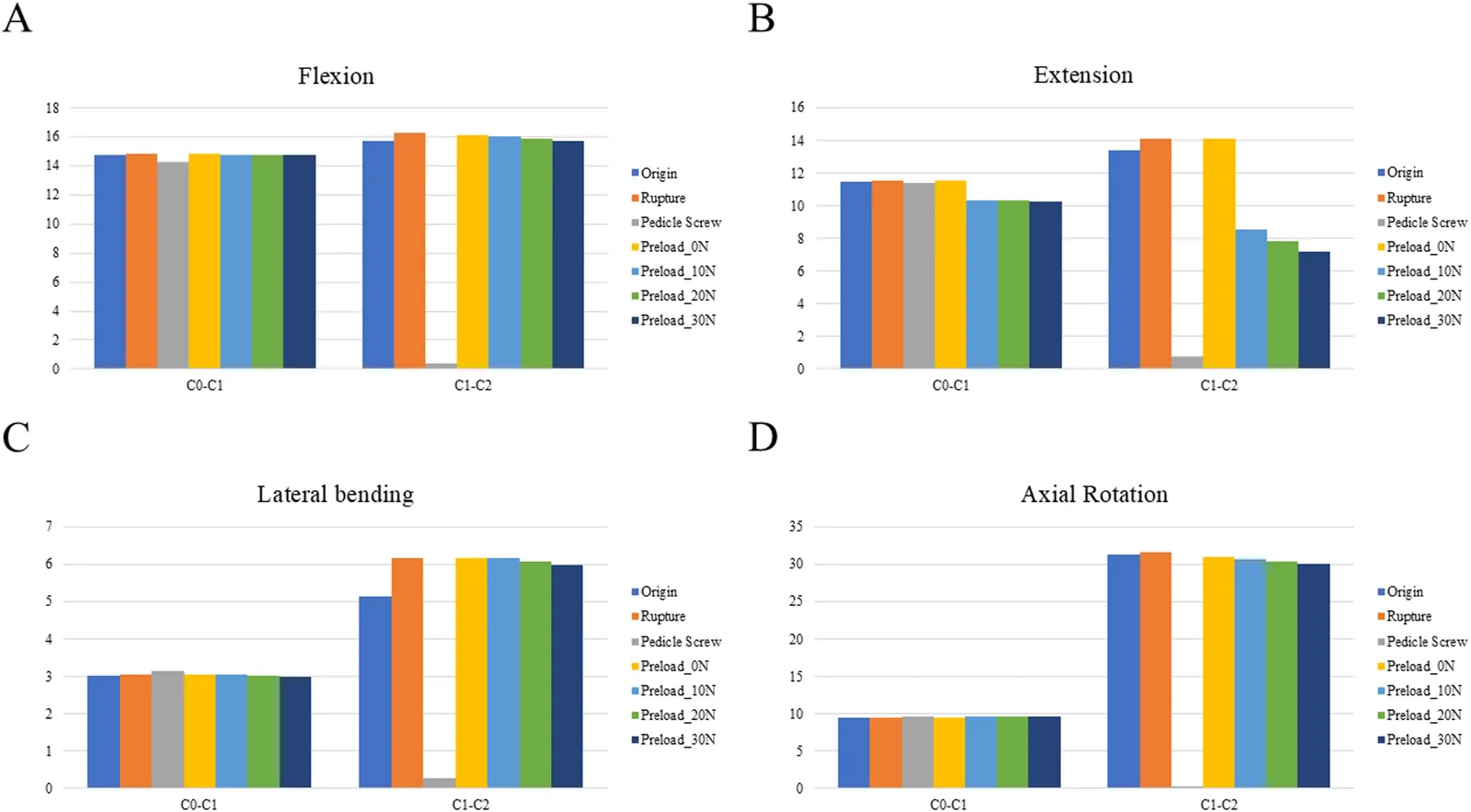
**A**-**D** ROM for flexion, extension, lateral bending, and axial rotation of C0–C1 and C1–C2 in each model.

Compared to the intact group, the C1–C2 ROM in all directions slightly increased in the TAL rupture group. However, compared to the intact group, the C1–C2 ROM in all directions significantly decreased in the posterior C1–C2 screw and rod fixation group. The C1–C2 ROM in all directions significantly increased in the ATAL reconstruction model without pretension compared to the posterior C1–C2 screw and rod fixation group. Compared to the intact group, the ATAL reconstruction model without pretension showed only a 2.43% increase in flexion, a 5.32% increase in extension, and a 20.82% increase in lateral bending, while axial rotation decreased by only 1.18%, effectively preserving the ROM of the C1–C2. Additionally, as the pretension gradually increased, the ROM in all directions of the TAL reconstruction group progressively decreased. For flexion and lateral bending, as the pretension increased, the ROM gradually approached that of the intact group, better preserving the ROM in these directions. In contrast, for axial rotation and extension, the opposite occurred. Specifically, when the pretension increased from 0 N to 10 N, the ROM in extension decreased sharply by 39.40%, significantly deviating from the ROM of the intact group compared to the no-pretension condition (Fig. 7).

## Discussion

The TAL is a crucial structure for maintaining the stability of the C1–C2. Its rupture can cause relative translation between C1 and C2, leading to AAI. This was well validated in our study, where after sectioning the TAL, both the AP displacement of C1–C2 and the ROM in all directions increased. The increase in AP displacement was the most significant, under a 100 N AP load, the relative translation distance increased from 2.842 mm before rupture to 8.283 mm after rupture, a sudden increase of 191.45%. On one hand, this aligns with Dickman et al.‘s report that “the atlantodental interval (ADI) in normal individuals should not exceed 3 mm, and values above 3 mm suggest TAL rupture,” further validating the reliability of our model [2]. On the other hand, it suggests that the primary function of the TAL is to restrict AP relative translation between the C1 and C2, with relatively minor effects on ROM. The abrupt increase in relative AP translation distance may lead to compression of the high cervical spinal cord within the spinal canal, potentially causing severe complications such as neurological deficits or even death [1]. Therefore, TAL rupture requires timely and appropriate treatment.

Treatment for TAL rupture varies depending on the classification of the rupture, and can be broadly categorized into conservative and surgical approaches. Patients who do not benefit from conservative treatment currently rely primarily on C1–C2 fusion or C0–C2 fusion for management. Although fusion surgeries restore stability to the atlantoaxial joint, they result in near-complete loss of mobility in the joint. In our posterior C1–C2 screw and rod fixation model, the AP displacement was only 0.227 mm, indicating that the fusion indeed restored stability to the atlantoaxial joint, but at the cost of significantly reduced ROM in all directions. The loss of atlantoaxial mobility can severely impact postoperative quality of life, as the atlantoaxial joint alone contributes nearly 50% of the cervical spine’s axial rotational ROM, and fusion surgeries are also associated with long-term complications such as accelerated degeneration of adjacent segments.

To address these issues, researchers have developed and validated various artificial atlantoaxial joints. In 2007, Hu Yong et al. [24], and in 2009, Lu Bin et al. [25], designed artificial atlantoaxial joints and evaluated them biomechanically. However, these designs only preserved the rotational function of the atlantoaxial joint and did not allow for flexion, extension, or lateral bending. In 2013, Cai Xuan et al. [7], and in 2021, Zang Quanjin et al. [5], improved upon these designs, enabling preservation of motion in all directions of the atlantoaxial joint. Additionally, in 2016, Chen Jinshui et al. [26], and in 2018, Shen Kai et al. [8], proposed a posterior dynamic fixation system for the atlantoaxial joint. With ongoing research and advancements in industry, both anterior and posterior artificial atlantoaxial joints have made significant strides in maintaining stability while preserving ROM. However, for the treatment of isolated TAL ruptures, the trauma and risks associated with joint replacement can be considered overtreatment to some extent. This scenario prompts us to draw parallels with knee replacements and cruciate ligament reconstruction, offering inspiration for TAL reconstruction.

Recently, Zhang Peng et al. [9] proposed an alternative method for reconstructing the TAL, which involves exposing the C1 lateral masses and the C2 spinous process, followed by drilling oblique and horizontal holes, respectively. However, the posterior and lateral aspects of the C1 lateral masses are in close proximity to the vertebral artery and vein, while the posterior and superior aspects of the C2 spinous process are blocked by the greater occipital nerve, lesser occipital nerve, and third occipital nerve. Avoiding these critical structures during surgery presents significant technical challenges. Additionally, to expose sufficient operative space, the surgical trauma would be considerable. Furthermore, there are anatomical variations between individuals in the C1 lateral masses and C2 spinous process. Ensuring consistent implantation outcomes in such a variable environment requires further investigation.

Therefore, we decided to use suture anchors to reconstruct the TAL at a location close to its original anatomical position. Our analysis of CT scans from 40 patients revealed a gap of approximately 4 mm between the odontoid process and the dura mater, and a gap of about 2 mm between the C1 lateral mass and the neck of the odontoid process. These spaces are sufficient to allow for surgical manipulation. Moreover, we can use an ultrasonic bone scalpel to slightly trim the narrow space formed by the C1 lateral mass, the anterior arch of the atlas, and the neck of the odontoid process, thereby moderately expanding the surgical field. To address the challenges of the confined surgical space and the risk of dura mater injury, we developed a specialized implantation instrument for the surgery. The distal part of the implantation forceps features a flexible channel that, similar to the function of a human thumb and index finger, can bend around the neck of the odontoid process while being pulled by “tendons” (traction threads). This design allows the channel to tightly envelop the neck of the odontoid process while occupying minimal space, thereby minimizing the risk of dura mater injury. This lays a solid foundation for the reconstruction of the TAL. However, the stability and functionality of the reconstructed atlantoaxial joint still require further validation.

Our study indicates that the non-pretensioned model of ATAL reconstruction shows a reduction of only 1.531 mm in AP displacement compared to the intact model, a significant decrease of 6.972 mm compared to the TAL rupture model, and an increase of 1.084 mm compared to the posterior C1–C2 screw and rod fixation model. These findings suggest that the reconstructed ligament possesses the ability to maintain the stability of the atlantoaxial joint similar to the original transverse ligament and fusion surgery.

In terms of ROM, the non-pretensioned ATAL model demonstrated increases of 2.43%, 5.32%, and 20.82% in flexion, extension, and lateral bending, respectively, compared to the intact model. Axial rotation was reduced by only 1.18%, effectively preserving the ROM of the atlantoaxial joint. The non-pretensioned ATAL model and the intact model showed minimal changes in ROM in all directions compared to the TAL rupture model. This may be attributed to the stabilizing role of auxiliary structures such as the contralateral TAL, VC, and the alar ligaments, which lessen the impact on the ROM of the atlantoaxial joint when a unilateral TAL is disrupted. When compared to the posterior C1–C2 screw and rod fixation model, the non-pretensioned ATAL model exhibited significant improvements in mobility in all directions. Overall, the reconstructed ATAL effectively restores and preserves both the stability and functional mobility of the atlantoaxial joint.

During our study, we also found that the pretension applied during the implantation of the ATAL significantly affects the reconstruction outcomes. Evaluating the pretension can provide guidance for achieving the appropriate tension during the surgical fixation of the ligament. As the pretension increased from 0 N in 10 N increments up to 30 N, the AP displacement in the ATAL reconstruction group gradually decreased, indicating that stability improved with increasing pretension. The ROM in flexion, extension, lateral bending, and axial rotation all decreased as the pretension increased. In flexion and lateral bending, the ROM of the atlantoaxial joint approached that of the intact model, while in extension and rotation, it moved further away from the intact model, especially in extension, where the ROM between C1–C2 decreased by nearly 40% at 10 N of pretension and further decreased with higher pretension. Notably, as the pretension increased, the friction between the ATAL and the odontoid process, as well as between the odontoid process and the anterior arch of the atlas, also increased. This could lead to wear and tear of the artificial ligament by the odontoid process, accelerating the failure of the artificial ligament, and potentially causing the odontoid process to be cut by the ligament. Additionally, the increased friction between the odontoid process and the anterior arch of the atlas may accelerate local joint degeneration and increase the risk of arthritis. Therefore, we believe that fixing the ATAL with minimal or no pretension during surgery may yield better outcomes.

To enhance the clinical applicability of the artificial reconstruction of the TAL, we have refined certain details of the procedure. In terms of implant selection, the suture anchors were utilized for the reconstruction of the TAL. The anchor portion is made of titanium alloy, which offers excellent biocompatibility and high strength. Its high-low double-thread design enhances its grip within the bone. The ultrabraid suture portion is made of ultra-high molecular weight polyethylene and polypropylene, which possess excellent characteristics such as high strength and wear resistance. These sutures are commonly used in orthopedic ligament repairs. Since the spinal canal contents of the atlas and axis include the high cervical spinal cord, to further enhance fault tolerance, we employed two pairs (yielding a total of four strands after folding) of ultrabraid sutures for the reconstruction of the TAL. In the event that one suture strand fails, the remaining strand can still provide stability. Moreover, the use of four ultrabraid sutures significantly improves the overall stiffness and contact area of the ATAL, reducing the friction and cutting effect of the sutures on the odontoid process. Lastly, despite the exceptional wear resistance of the ultrabraid sutures, it is still crucial to ensure that the threaded holes at the anchors’ tail, which hold the sutures, align with the direction of the suture path, and maintain an angle of more than 90° between the anchor and the suture during fixation. This alignment minimizes the risk of cutting between the anchor and the suture, thereby extending the lifespan of the implant, reducing the risk of postoperative implant failure, and minimizing the production of frictional debris. This approach also decreases the likelihood of sterile inflammatory reactions caused by local foreign body irritation.

Ensuring the stability of the reconstructed atlantoaxial joint after the implantation of the ATAL is equally crucial during surgery. The method proposed by Andy Ottenbacher et al. [27] effectively addresses this challenge by having the patient positioned prone with the head secured in a Mayfield clamp. The surgeon then uses two Kocher clamps to grip the posterior arch of the atlas and the spinous process of the axis, stabilizing C1. With an upward pull of approximately 50 N applied to C2, fluoroscopy is utilized to closely monitor whether the ADI exceeds 5 mm. Additionally, fixing C2 and tilting C1 downward can reveal instability if tilting occurs between C1 and C2 and an anterior gap is observed. This approach underscores the importance of indirect assessments like the ADI in evaluating the integrity of the TAL.

It must be acknowledged that our study has several limitations. First, an isolated TAL rupture represents the optimal indication for the TAL reconstruction technique. However, TAL rupture often occurs in combination with other injuries, such as atlas fractures, rather than in isolation. Therefore, the clinical applicability of TAL reconstruction may be limited by the relatively small number of suitable patients. Combining necessary fixation techniques with TAL reconstruction could expand the indications for this procedure. In our opinion, as long as the internal fixation does not block the anchor entry point into the lateral mass of the atlas, any fixation technique can be combined with TAL reconstruction. If the chosen internal fixation unavoidably conflicts with the anchor placement for TAL reconstruction, a staged procedure may be worth considering. Notably, when anterior open reduction and internal fixation of an atlas fracture is performed, we believe the anchor screws used for TAL reconstruction could be redesigned – for example, by configuring the tail thread of the anchor to match the locking hole of a plate. This would allow the same anchor to both fix the plate and serve as the attachment point for the ultrabraid sutures, thereby providing a more comprehensive solution for an unstable TAL rupture combined with an atlas fracture. This approach appears promising, and we plan to explore it further in future studies. For instance, in an anterior approach, using the Jefferson‑fracture reduction plate (JeRP) designed by Qiang Tu et al. [28], the lateral‑mass screws could be replaced with locking anchors, allowing simultaneous treatment of the atlas fracture and TAL rupture.

Second, we chose a transoral approach for the surgery. This approach has inherent risks, including a limited operative field and a relatively high infection risk [29]. Moreover, TAL rupture may be associated with other injuries requiring additional internal fixation procedures, which would increase surgical trauma and operative time, further raising the infection risk when combined with a transoral approach. To mitigate these risks, perioperative antibiotic prophylaxis can be administered according to guidelines, rigorous sterilization should be performed before and after surgery, the wound can be soaked with povidone‑iodine for 15 min before closure, the surgery should be performed by an experienced surgeon to shorten operative time, and soft tissues should be carefully protected [28, 30]. In addition, if the tip of the lateral‑mass anchor protrudes too far, it may cause posterior pharyngeal wall wound dehiscence or postoperative dysphagia [28]. We should minimize the exposed portion of the anchor tail outside the lateral mass of the atlas. Finally, we used ultrabraid sutures to reconstruct the TAL. These sutures require long‑term relative motion against the posterior surface of the odontoid neck. In orthopaedics, it is uncommon for a non‑biological reconstruction material to sustain continuous dynamic contact with a non‑articular bony surface in the absence of a dedicated cartilage or bearing interface. In this regard, we note that the atlanto‑dental joint is a non‑weight‑bearing joint. When no additional preload is applied, there is no extra compressive load between the odontoid process and the reconstruction material. Furthermore, to reduce the cutting effect of the ultrabraid sutures on the odontoid process, we used four strands of suture to increase the contact area. From another perspective, the odontoid process is primarily loaded during anterior‑posterior translation and flexion of the atlantoaxial joint. We analysed these two scenarios separately: During anterior‑posterior translation, the reconstructed TAL is positioned slightly lower than the original TAL. Therefore, according to the lever principle, the moment arm on the odontoid process is shorter. For the same anterior‑posterior force, the stress concentration at the base of the odontoid process is reduced, making odontoid fracture less likely. The same reasoning applies during flexion. Additionally, some studies have suggested that the secondary stabilising structure – the C0‑C1‑C2 ligament complex – may provide sufficient atlantoaxial stability even when the TAL is insufficient [31]. If so, the odontoid process might not need to bear a large load from the reconstruction material. Of course, to further evaluate the real‑world clinical applicability of this technique, cadaveric biomechanical experiments are warranted in the future.

## Conclusions

With the assistance of an ATAL implant instruments, the ruptured TAL can be reconstructed at approximately its original anatomical position. Additionally, the reconstructed ATAL has the ability to restore stability in the atlantoaxial joint while preserving its ROM. Furthermore, applying no or minimal pre-tension when fixing the TAL may lead to better clinical outcomes.

## Data Availability

The datasets used and/or analysed during the current study are available from the corresponding author on reasonable request.

## Abbreviations

TAL: Transverse atlantal ligament
AAI: Atlantoaxial instability
ROM: Range of motion
ATAL: Artificial transverse atlantal ligament
FEA: Finite element analysis
CT: Computed tomography
AF: Annulus fibrosus
VC: Vertical cruciate
LAO: Lateral atlantooccipital ligament
LF: Ligamentum flavum
CL: Capsular ligament
ISL: Interspinous ligaments
AAOM: Anterior atlantoaxial ligament
PAOM: Posterior atlantoaxial ligament
TM: Tectorial membrane
AP: Anterior-posterior
SD: Standard deviation
ADI: Atlantodental interval
